# Comparative studies between the murine immortalized brain endothelial cell line (bEnd.3) and induced pluripotent stem cell-derived human brain endothelial cells for paracellular transport

**DOI:** 10.1371/journal.pone.0268860

**Published:** 2022-05-25

**Authors:** Jiahong Sun, Weijun Ou, Derick Han, Annlia Paganini-Hill, Mark J. Fisher, Rachita K. Sumbria

**Affiliations:** 1 Department of Biomedical and Pharmaceutical Sciences, School of Pharmacy, Chapman University, Irvine, CA, United States of America; 2 Department of Biopharmaceutical Sciences, School of Pharmacy and Health Sciences, Keck Graduate Institute, Claremont, CA, United States of America; 3 Department of Neurology, University of California, Irvine, Irvine, CA, United States of America; 4 Department of Pathology & Laboratory Medicine, University of California, Irvine, Irvine, CA, United States of America; Eötvös Loránd Research Network Biological Research Centre, HUNGARY

## Abstract

Brain microvascular endothelial cells, forming the anatomical site of the blood-brain barrier (BBB), are widely used as *in vitro* complements to *in vivo* BBB studies. Among the immortalized cells used as *in vitro* BBB models, the murine-derived bEnd.3 cells offer culturing consistency and low cost and are well characterized for functional and transport assays, but result in low transendothelial electrical resistance (TEER). Human-induced pluripotent stem cells differentiated into brain microvascular endothelial cells (ihBMECs) have superior barrier properties, but the process of differentiation is time-consuming and can result in mixed endothelial-epithelial gene expression. Here we performed a side-by-side comparison of the ihBMECs and bEnd.3 cells for key paracellular diffusional transport characteristics. The TEER across the ihBMECs was 45- to 68-fold higher than the bEnd.3 monolayer. The ihBMECs had significantly lower tracer permeability than the bEnd.3 cells. Both, however, could discriminate between the paracellular permeabilities of two tracers: sodium fluorescein (MW: 376 Da) and fluorescein isothiocyanate (FITC)–dextran (MW: 70 kDa). FITC-dextran permeability was a strong inverse-correlate of TEER in the bEnd.3 cells, whereas sodium fluorescein permeability was a strong inverse-correlate of TEER in the ihBMECs. Both bEnd.3 cells and ihBMECs showed the typical cobblestone morphology with robust uptake of acetylated LDL and strong immuno-positivity for vWF. Both models showed strong claudin-5 expression, albeit with differences in expression location. We further confirmed the vascular endothelial- (CD31 and tube-like formation) and erythrophagocytic-phenotypes and the response to inflammatory stimuli of ihBMECs. Overall, both bEnd.3 cells and ihBMECs express key brain endothelial phenotypic markers, and despite differential TEER measurements, these *in vitro* models can discriminate between the passage of different molecular weight tracers. Our results highlight the need to corroborate TEER measurements with different molecular weight tracers and that the bEnd.3 cells may be suitable for large molecule transport studies despite their low TEER.

## Introduction

The brain microvascular endothelial cells form the anatomical site of the blood-brain barrier (BBB) [1]. Experimental evidence showing the presence of the BBB dates to more than 100 years ago, wherein simple vital dyes were used to show the presence of a barrier separating the peripheral circulation and the central nervous system [2]. The primary site of the barrier is the endothelial layer lining the cerebral microvasculature, which is further supported by the presence of a basement membrane lining the endothelial cells and the pericytes, and the astrocyte foot-processes that envelope 99% of the cerebral microvasculature [3]. The key features of the brain microvascular endothelial cells that make the BBB an impermeable interface are the continuous arrangement of tight-junction proteins, lack of fenestrae, low pinocytic activity, and the resultant high transendothelial electrical resistance (TEER). As a result, even small molecule transport, which is restricted to molecules < 400 Da that have < 3 hydrogen bond donors and < 7 hydrogen bond acceptors, is limited at the BBB [4–6]. Other features that make the BBB a highly selective physical barrier include high mitochondrial content and polarized expression of transporters and metabolic enzymes [7, 8]. Additionally, the brain microvascular endothelial cells respond to inflammatory stimuli through the expression of adhesion molecules and exhibit vascular endothelial characteristics [9]. These peculiar characteristics are required for the optimal functioning of the BBB to maintain a stable brain microenvironment.

The BBB is altered in some pathological conditions [1] and is the primary obstacle to brain drug delivery [10]. In this respect, *in vitro* BBB models are useful complements to *in vivo* studies and have increased our understanding of normal BBB function, BBB alterations under pathological conditions, and drug transport [11]. Brain microvascular endothelial cells from different species (human and non-human) have been widely used as *in vitro* BBB models and include primary brain microvascular endothelial cells and immortalized cells lines [12]. Though validated for key phenotypic attributes, one of the major challenges in using these models has been achieving the high electric resistance observed *in vivo* in humans (~8000 ohm·cm^2^) [11–13]. This major limitation has been overcome with the use of human-induced pluripotent stem cells (iPSCs)-derived human brain microvascular endothelial cells (ihBMECs), which was first reported in 2012 [14]. Since then, several research groups have differentiated iPSCs into ihBMECs, which consistently have superior barrier properties compared with the existing primary and immortalized human and non-human mammalian cell models [15–17]. However, iPSC differentiation can result in a heterogeneous cell population, and recent transcriptomic studies have demonstrated that ihBMECs express genes that are more consistent with a neuroectodermal epithelial lineage than an endothelial-vascular lineage [9]. Therefore, ihBMECs are now reported to have a mixed endothelial-epithelial transcriptional profile and are largely classified as ihBMEC-like cells [9, 18, 19]. Further, despite the superior BBB characteristics of the ihBMECs, the process of differentiating human iPSCs into ihBMEC-like cells is time-consuming, expensive, and can result in batch-to-batch variability in the quality of the differentiated cells requiring rigorous phenotypic confirmation [20]. In this respect, using immortalized cell lines has the advantage of culturing consistency, low cost, and maintenance of phenotypic features over several passages [12].

Among the immortalized cell lines used for BBB studies *in vitro*, the murine-derived bEnd.3 cell line is one of the most widely used and well-characterized for functional and transport assays [21–24]. Since *in vivo* murine models are extensively used in preclinical research, the use of a murine-derived cell line complements *in vivo* murine studies. To our knowledge, no side-by-side comparison of the bEnd.3 cells and ihBMEC-like cells has been performed. Therefore, in the current study, we performed a side-by-side comparison of the ihBMEC-like cells and bEnd.3 cells with respect to the following key paracellular transport characteristics at the BBB: barrier function (TEER and tight-junction protein expression) and paracellular permeability of different molecular weight markers–sodium fluorescein and fluorescein isothiocyanate (FITC)–dextran. Additionally, the endothelial phenotype of the cells was confirmed by their cobblestone morphology, expression of endothelial marker von Willebrand factor (vWF), and uptake of acetylated low-density lipoprotein (LDL). The vascular endothelial phenotype of the ihBMECs was supported by the expression of CD31 and evidence of tube-like structures, and response to inflammatory stimuli. Finally, to gain insight into the suitability of the ihBMECs to model blood cell interactions, we studied erythrophagocytosis of aged red blood cells, a phagocytic phenotype that is now increasingly reported for vascular endothelial cells [25–30].

## Methods

### Murine brain microvascular endothelial cell culture

bEnd.3 cells (American Type Culture Collection, Manassas, VA, USA; Catalog no. CRL-2299) were cultured in Dulbecco’s Modified Eagle’s Medium (American Type Culture Collection, Manassas, VA, USA) supplemented with 10% fetal bovine serum (R&D systems, Minneapolis, MN, USA), and 100 μg/mL penicillin/streptomycin (Sigma-Aldrich, St. Louis, MO, USA) at standard cell culture conditions (5% CO_2_, 95% air). Cells between passages 24 and 30 were seeded onto multi-well plates (Corning Inc., Corning, NY, USA), 0.2% gelatin-coated glass coverslips, or Transwells with 0.4 μm pore polyester membrane inserts of 24-well plates (Corning Inc., Corning, NY, USA) at a density of 1x10^5^ to 1x10^6^ cells/cm^2^, unless otherwise stated.

### Human iPSC culture and differentiation

Human iPSC IMR90-4 line (WiCell, Madison, WI, USA, passage 34–37; Catalog no. WISCi004-B) was differentiated into ihBMECs as described previously [31]. Briefly, IMR90-4 cells were maintained on Matrigel (Corning Inc., Corning, NY, USA)–coated surfaces in mTeSR-plus (STEMCELL Technologies, Vancouver, BC, Canada). Before differentiation, iPSCs were singularized with Accutase (Innovative Cell Technologies, San Diego, CA, USA) and plated onto Matrigel-coated 6-well plates at a density of 2.5 x 10^4^ cells/cm^2^ in mTeSR-plus supplemented with 10 μM ROCK inhibitor Y-27632 (Selleckchem, Houston, TX, USA). After a 3-day culture in mTeSR-plus, differentiation was initiated by treating cells with 6 μM CHIR99021 (Selleckchem, Houston, TX, USA) in DMEM/Ham’s F12 (Thermo Fisher, Waltham, MA, USA) with 1X MEM nonessential amino acids (Thermo Fisher, Waltham, MA, USA), 0.5X GlutaMAX (Thermo Fisher, Waltham, MA, USA), and 0.1 mM β-mercaptoethanol (Sigma-Aldrich, St. Louis, MO, USA) for 1 day. Cells were re-fed 2 mL per well of DMEM/Ham’s F12 (Thermo Fisher, Waltham, MA, USA) with 1X MEM nonessential amino acids (Thermo Fisher, Waltham, MA, USA), 0.5X GlutaMAX (Thermo Fisher, Waltham, MA, USA), and 0.1 mM β-mercaptoethanol (Sigma-Aldrich, St. Louis, MO, USA) every 24 h for five days. The medium was changed to human Endothelial Serum-Free Medium (hESFM, Invitrogen, Carlsbad, CA, USA) supplemented with 20 ng/mL bFGF, 1X B27 (Thermo Fisher, Waltham, MA, USA), and 10 μM retinoic acid (Sigma-Aldrich, St. Louis, MO, USA) for three days. Cells were dissociated with Accutase and re-plated at 1 x 10^6^ cells/cm^2^ in the same medium onto coverslips, or 24-well Transwell inserts (pore size 0.4 μm, Corning Inc., Corning, NY, USA) coated with a mixture of collagen IV (400 μg/mL; Sigma-Aldrich, St. Louis, MO, USA) and fibronectin (100 μg/mL; Sigma-Aldrich, St. Louis, MO, USA). At 24 h after the re-plating, ihBMECs were cultured in hESFM with 1X B27 for culture and replaced with fresh medium daily. For initial experiments, monolayers growing on Transwell inserts were stained with hematoxylin and eosin (H&E), as described previously [27, 32], to confirm monolayer formation on the Transwell inserts.

### TEER measurement

bEnd.3 cells or ihBMECs at a density of 1 x 10^5^ to 1 x 10^6^ cells/cm^2^ were grown on 24-well Transwell inserts (pore size 0.4 μm, Corning Inc., Corning, NY, USA). The integrity of brain endothelial monolayer in Transwells inserts was assessed by measuring TEER using the EVOM2 Epithelial Volt/Ohm Meter and an STX-2 electrode system (World Precision Instruments LLC, Sarasota, FL, USA) for five days. ihBMECs were replenished with fresh medium daily, and medium change for bEnd.3 cells was performed every two days. TEER measurements were corrected by subtracting the TEER of cell-free (empty) Transwells. TEER is reported in Ω x cm^2^ and % of baseline.

### Permeability of FITC-dextran and sodium fluorescein

In a subset of experiments, bEnd.3 cells or ihBMECs were cultured onto 24-well Transwell inserts (pore size 0.4 μm, Corning Inc., Corning, NY, USA) until TEER was stabilized (usually 72 to 96 h after seeding). Sodium fluorescein (5 μg/mL, MW: 376 Da, Sigma-Aldrich, St. Louis, MO, USA) or FITC-dextran (250 μg/mL, MW: 70 kDa, Sigma-Aldrich, St. Louis, MO, USA) was added to the medium in the apical chamber every 24 h starting two days after seeding the cells. After tracer addition to the apical chamber, medium in the basolateral chamber was collected every 24 h for up to three days, and the mean fluorescent intensity (MFI) of sodium fluorescein and FITC-dextran was measured using a fluorescence plate reader (Molecular Devices, LLC, San Jose, CA, USA) at an excitation/emission wavelength of 460 nm/515 nm and 490 nm/520 nm, respectively. The permeability of FITC-dextran and sodium fluorescein across the brain endothelial monolayer is reported as the permeability coefficient calculated based on the Fick’s law as described previously [33]: Permeability coefficient (P, cm/s) = [volume of basolateral chamber / (surface area of Transwells x the initial concentration of apical chamber)] × [the diffusion concentration of basolateral chamber / diffusion time]. To determine the ability of the endothelial monolayer to block tracer passage, we compared the percentage of the applied tracer in the basolateral Transwell across the endothelial monolayer to that across cell-free Transwell inserts. Transport of FITC-dextran and sodium fluorescein across the cell-free Transwells was used as a measure of spontaneous migration, and tracer passage across the endothelial monolayer is reported as % of spontaneous migration.

### Acetylated LDL uptake

bEnd.3 cells were cultured on gelatin-coated coverslips, and ihBMECs were grown on collagen/fibronectin-coated coverslips. Before adding acetylated LDL, cells were incubated with medium supplemented with 0.3% bovine serum albumin (BSA, Fisher Scientific, Waltham, MA, USA) for 1 h at 37°C to block non-specific binding. Subsequently, bEnd.3 cells and ihBMECs were incubated with 10 μg/mL Alexa Fluor 488-conjugated acetylated LDL (Invitrogen, Carlsbad, CA, USA) for 4 h at 37°C followed by three PBS washes. After fixation with 4% paraformaldehyde, the nucleus was stained with DRAQ5 (Biolegend, San Diego, CA, USA), and coverslips were mounted with UltraCruz Aqueous Mounting Medium (Santa Cruz Biotechnology, Dallas, TX, USA). Three random fields per coverslip (of approximately 50 cells each) were imaged using a 63x oil immersion objective with a Leica SP5 confocal microscope (Leica, Wetzlar, Hessen, Germany) and manually quantified for cells positive with acetylated LDL uptake. iPSC IMR90-4 cells growing on Matrigel-coated coverslips were stained for acetylated LDL uptake as a phenotype negative control.

### vWF, claudin-5, and CD31 immunofluorescence

bEnd.3 cells and ihBMECs growing on coverslips were fixed with 4% paraformaldehyde and permeabilized with 0.2% Triton X-100 (Fisher Scientific, Waltham, MA, USA). Cells were further blocked with a blocking buffer containing 1% bovine serum albumin (Fisher Scientific, Waltham, MA, USA), 0.1% Tween 20 (Bio-Rad, Hercules, CA, USA), and 22.5 mg/mL glycine (Sigma-Aldrich, St. Louis, MO, USA). Cells were incubated with monoclonal mouse anti-claudin-5 antibody (Invitrogen, Carlsbad, CA, USA, 1:100 in blocking buffer; Catalog no. 35–2500), polyclonal rabbit anti-vWF antibody (Agilent Technologies, Santa Clara, CA, USA, 1:100 in blocking buffer; Catalog no. A0082) or polyclonal rabbit anti-CD31 antibody (Abcam, Cambridge, MA, USA, 1:100 in blocking buffer; Catalog no. 28364) overnight at 4°C. Anti-claudin-5 antibody was detected using the mouse-IgGk BP-CFL 488 binding protein (Santa Cruz Biotechnology, Dallas, TX, USA, 1:100 in blocking buffer; Catalog no. 516176), while anti-vWF and anti-CD31 antibodies were detected using the Alexa Fluor 488 labeled goat anti-rabbit IgG H&L secondary antibody (Abcam, Cambridge, MA, USA, 1:100 in blocking buffer; Catalog no. 150077). Cell nuclei were visualized by DRAQ5 staining, and coverslips were mounted with the UltraCruz Aqueous Mounting Medium (Santa Cruz Biotechnology, Dallas, TX, USA). Three random fields (of approximately 50 cells each) per coverslip were imaged using a 63x oil immersion objective with a Leica SP5 confocal microscope (Leica Wetzlar, Hessen, Germany). Z-Stacks were acquired with a z-step size of 0.5 μm, and maximum projection images were used to quantify vWF and claudin-5 expression using NIH ImageJ (NIH, Baltimore, M, USA). For vWF quantification, cells were manually counted, and the percentage of cells positive for vWF was calculated. For claudin-5 quantification, the MFI of claudin-5 was standardized based on the cell number and reported as % of bEnd.3 cells.

### Response of ihBMECs to inflammatory stimuli

ihBMECs at a density of 1 x 10^6^ cells/cm^2^ were grown on 24-well Transwell inserts (pore size 0.4 μm, Corning Inc., Corning, NY, USA) that were coated with a mixture of collagen IV (400 μg/mL; Sigma Aldrich, MO, USA) and fibronectin (100 μg/mL; Sigma Aldrich, MO, USA) in human Endothelial Serum-Free Medium (hESFM, Invitrogen, CA, USA) supplemented with 20 ng/mL bFGF, 1x B27 (Thermo Fisher Scientific, MA, USA) and 10μM retinoic acid (Sigma Aldrich, MO, USA) for 24 h. ihBMECs were cultured in hESFM with 1x B27 for an additional three days with daily media change, after which the cells were treated with lipopolysaccharide (LPS) from Salmonella enterica serotype typhimurium (100 μg/mL, Sigma-Aldrich, St. Louis, MO, USA) or tumor necrosis factor-alpha (TNFα, 1 μg/mL) for 48 h with daily media change. The integrity of brain endothelial monolayer in Transwell inserts was assessed by measuring TEER, which was corrected by subtracting the TEER of cell-free (empty) Transwells. TEER is reported as % of baseline.

### Erythrophagocytic phenotype of ihBMECs

Human red blood cells in Alsever’s solution were obtained from healthy male volunteers aged 28–29 years (BioIVT, New York, NY, USA). Red blood cells were treated either with sterile phosphate-buffered saline (PBS, Invitrogen, Waltham, MA, United States) as control or 3 mM tert-butylhydroperoxide (tBHP, Sigma-Aldrich, St. Louis, MO, United States) at 37°C for 30 min. tBHP is an oxidative stressor used to expose phosphatidylserine on the outer leaflet of the red blood cell membrane to mimic aged red blood cells *in vitro* [27]. PBS- and tBHP-treated red blood cells (5 x 10^6^) were incubated with ihBMECs seeded onto collagen and fibronectin-coated coverslips in a 24-well plate for 48 h. Cells were stained with H&E to detect attachment and engulfment of red blood cells by ihBMECs as described previously [27], and H&E-stained slides were imaged using a confocal microscope to confirm red blood cell internalization.

### Statistical analysis

The data are shown as means ± S.E.M of 3–8 independent biological repeats (cells from different passages for bEnd.3 cells and ihBMECs obtained from independent iPSC differentiation runs). Unpaired Student’s t-test was used to compare two independent groups. To test the effect of two factors, two-way repeated-measures ANOVA with Holm-Sidak post hoc test was used. Correlation between numerical variables was performed using the Pearson correlation. GraphPad Prism 8.0 (GraphPad Software, Inc., La Jolla, CA, USA) was used for statistical analyses, and a two-tailed p< 0.05 was considered statistically significant.

## Results

### TEER measurement across the bEnd.3 and ihBMEC monolayer

**Fig 1:**
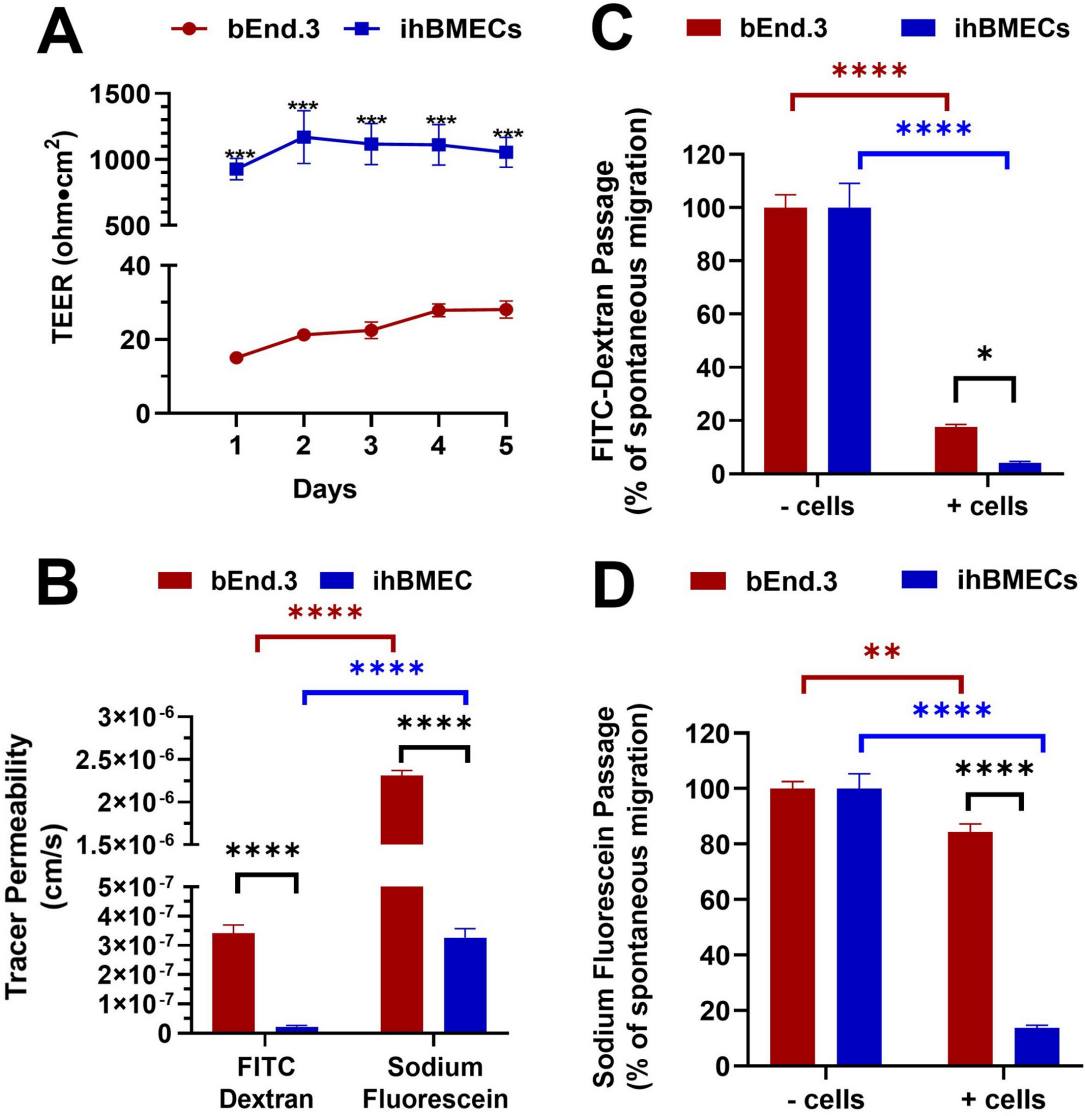
Transendothelial electrical resistance (TEER) and tracer passage. TEER expressed in ohm.cm^2^ (**A**). Permeability of FITC-dextran and sodium fluorescein (**B**) across the bEnd.3 and ihBMEC monolayers grown on Transwell inserts. Reduction in the spontaneous passage of FITC-dextran (**C**) and sodium fluorescein (**D**) by the bEnd.3 and ihBMEC monolayers. Spontaneous migration represents the transport of the tracer in the absence of the brain endothelial monolayer. Data are presented as mean ± SEM of n = 3–8 per time point. *p<0.05, **p<0.01, ***p<0.001, ****p<0.0001, by two-way ANOVA and Holm-Sidak post hoc test.

To compare the paracellular passage across the brain endothelial monolayers, we performed TEER measurements starting 24 h after seeding cells on Transwells. The average TEER of the cell-free inserts was 41 ± 0.5 ohm·cm^2^. TEER values of cell-free inserts were subtracted from the TEER values of inserts with endothelial cells and are shown in Fig 1A. As shown in Fig 1A, over the five days, the TEER values across the bEnd.3 monolayer ranged between 15 ± 0.8 ohm·cm^2^ (day 1) and 28.1 ± 2.3 ohm·cm^2^ (day 5). The TEER measurements across the ihBMECs were at least 42-fold higher than those across the bEnd.3 monolayer and ranged between 928 ± 81 ohm·cm^2^ (day 1) and 1169 ± 200 ohm·cm^2^ (day 2) (Fig 1A). Many studies using bEnd.3 cells have reported TEER measurements for 6–8 days [34–37]. However, our pilot studies showed that TEER measurements across the ihBMECs begin to decline five days after replating on Transwells inserts (TEER values on day 6 were: 796 ± 46 ohm cm^2^). Therefore, TEER measurements were performed for five days in the current study.

### Permeability of sodium fluorescein and FITC-dextran across the bEnd.3 and ihBMEC monolayer

In addition to reporting TEER as a measure of paracellular transport, we measured the transport of two molecular weight tracers: sodium fluorescein (MW: 376 Da) and FITC-dextran (MW: 70 kDa), across the brain endothelial monolayers. The aim was to determine the ability of the brain endothelial monolayers to restrict tracer passage and determine if tracer permeability paralleled TEER measurements. The permeability of FITC-dextran and sodium fluorescein across the bEnd.3 monolayer was 3.4 ± 0.3 x 10^−7^ cm/s and 2.3 ± 0.06 x 10^−6^ cm/s, respectively (Fig 1B). The permeability of FITC-dextran and sodium fluorescein across the ihBMEC monolayer was an order of magnitude lower at 2.2 ± 0.4 x 10^−8^ cm/s and 3.3 ± 0.3 x 10^−7^ cm/s, respectively, than that across the bEnd.3 monolayer (Fig 1B). Twenty-four hours after incubation, the driving force for tracer diffusion was confirmed by calculating the ratio of the apical to basolateral tracer concentration. The apical tracer concentration was at least 17-fold higher than the basolateral tracer concentration across the bEnd.3 and ihBMEC monolayer except for sodium fluorescein, which passed rather freely across the bEnd.3 monolayer (apical to basolateral concentration ratio ranged between 1 to 4).

We also compared the ability of the two brain endothelial monolayers to restrict the spontaneous passage of these tracers. This was done by measuring the percentage of the applied tracer transported across the endothelial monolayers and comparing that to the percentage of the applied tracer transported across cell-free Transwells. The bEnd.3 monolayer restricted the free-passage of FITC-dextran (free passage is the passage of the fluorescent tracer in the absence of the endothelial monolayer) by >80%, while the ihBMECs restricted the free passage of FITC-dextran by >90% (Fig 1C). However, the bEnd.3 monolayer restricted the free passage of sodium fluorescein by only ~15%, while the ihBMECs restricted the free passage of sodium fluorescein by ~87% (Fig 1D).

**Fig 2:**
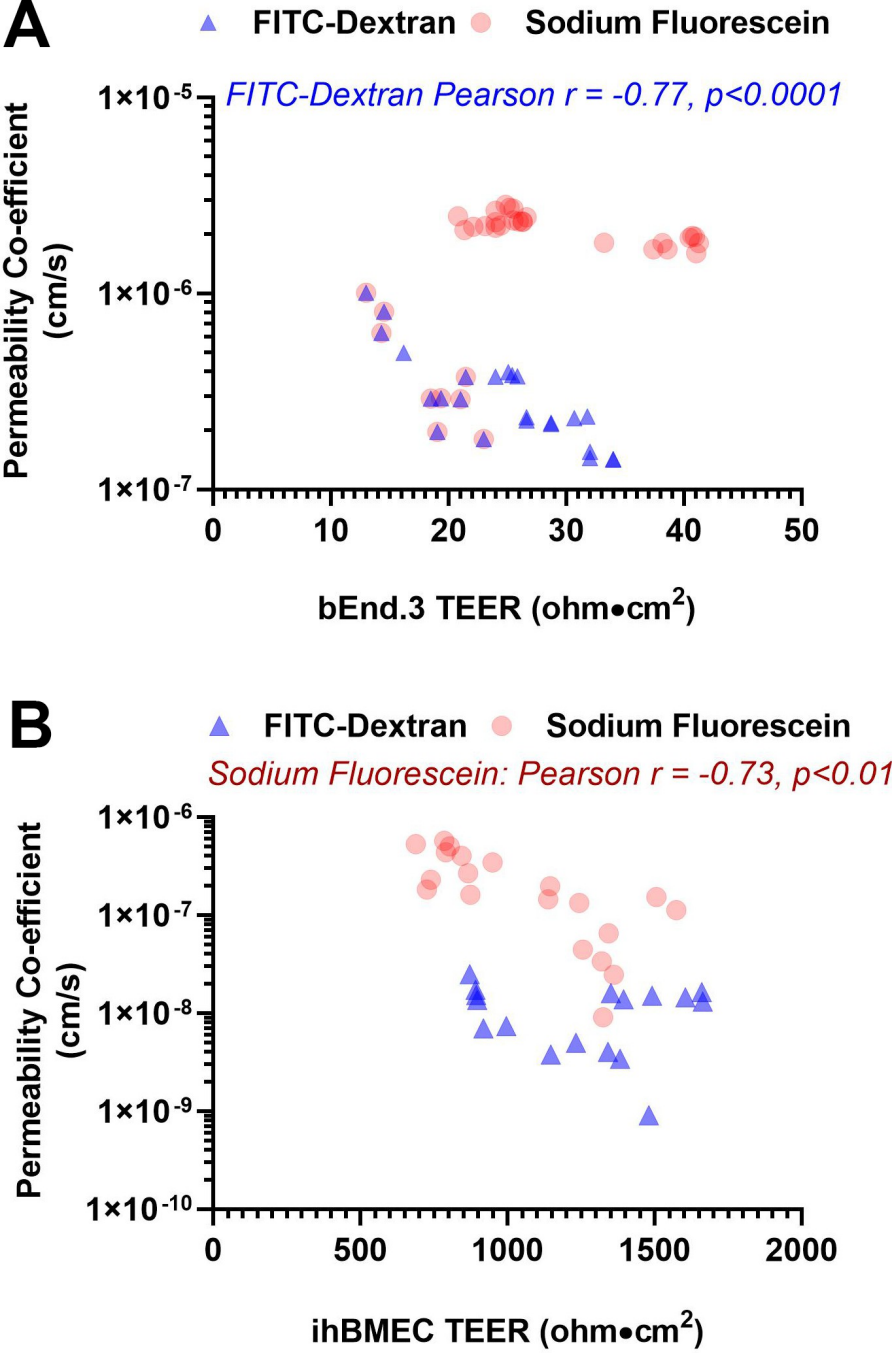
Correlation between TEER measurements and tracer permeability across the bEnd.3 and ihBMEC monolayer. A significant inverse correlation was observed between FITC-dextran and TEER using the bEnd.3 cells (*Pearson r* = -0.77, p<0.0001) (**A**), and between sodium fluorescein and TEER while using the ihBMECs (*Pearson r* = -0.73, p<0.01) (**B**).

We found a strong inverse correlation between FITC-dextran permeability and TEER measurements across the bEnd.3 monolayer (Pearson r = -0.77, p<0.0001), but the permeability of sodium fluorescein across the bEnd.3 monolayer did not correlate with TEER measurements (Pearson r = 0.32, not significant (NS)) (Fig 2A). In contrast, sodium fluorescein permeability showed a strong inverse correlation with the TEER measurements across the ihBMEC monolayer (Pearson r = -0.73, p<0.01), while no significant correlation was observed between the permeability of FITC-dextran and TEER across the ihBMEC monolayer (Pearson r = -0.16, NS) (Fig 2B).

### Endothelial phenotypic marker and tight junction protein expression

**Fig 3:**
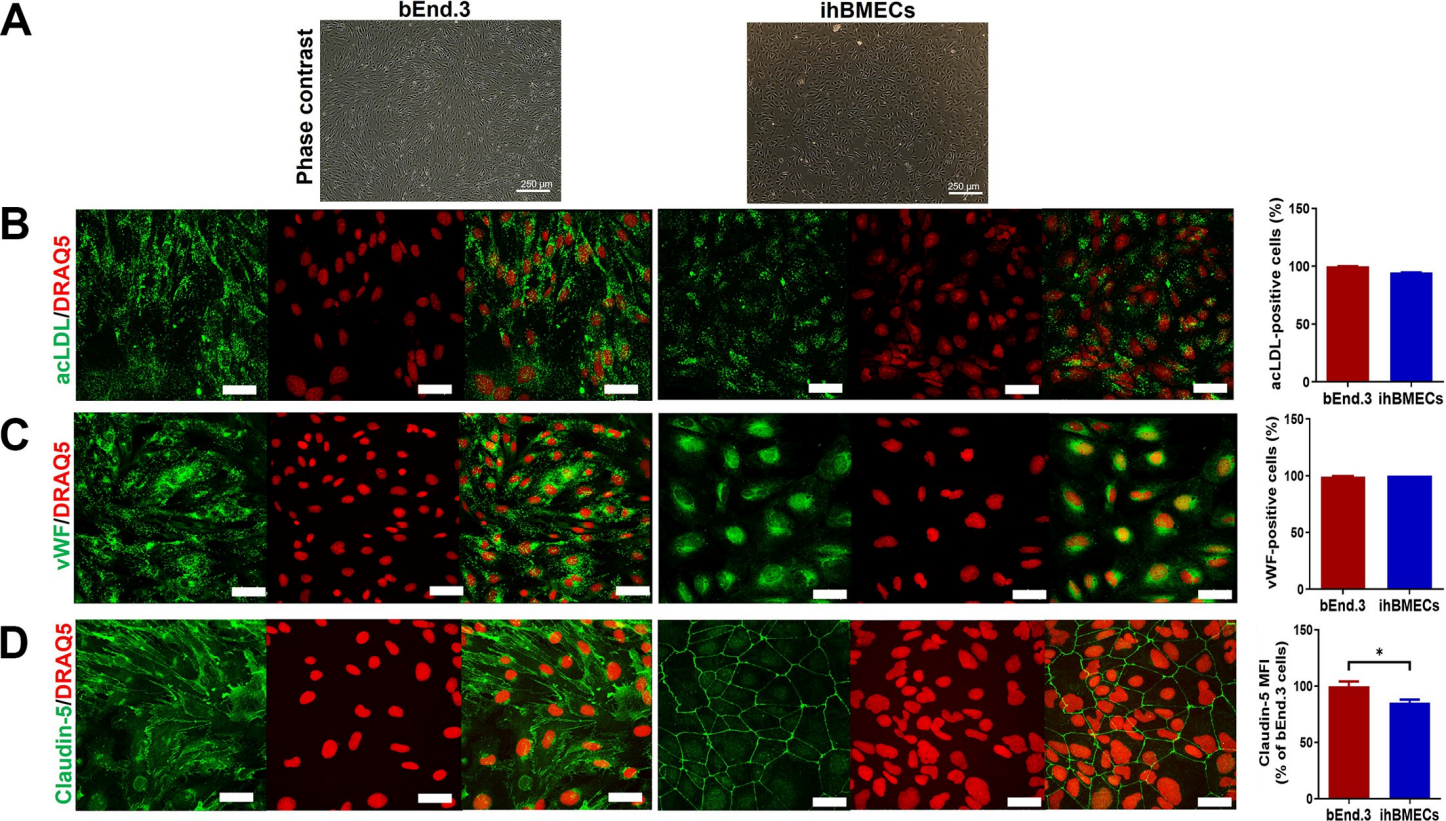
Endothelial phenotypic confirmation of bEnd.3 cells and ihBMECs. Phase-contrast images showing the typical cobblestone morphology of the bEnd.3 and ihBMECs (**A**, scale bar = 250 μm). Uptake of AlexaFluor-488 labeled acetylated low-density lipoprotein (acLDL) by the bEnd.3 cells and ihBMECs (shown as green in **B**). Immunofluorescent detection of Von Willebrand factor (vWF) (shown as green in **C**) and claudin-5 (shown as green in **D**) in bEnd.3 cells and ihBMECs. The nucleus is stained with DRAQ5 (shown as red). Scale bar = 40 μm. Data are presented as mean ± SEM of n = 3 independent repeats. *p<0.05 by Student’s t-test.

Next, we wanted to compare the expression of key endothelial phenotypic markers and tight junction protein expression between the two brain endothelial monolayers. The phase-contrast images of the bEnd.3 cells and ihBMECs (Fig 3A) show the typical cobblestone morphology, observed for endothelial and epithelial cells [38, 39]. Both bEnd.3 cells and ihBMECs showed robust uptake of acetylated LDL (Fig 3B) and strong immuno-positivity for vWF (Fig 3C). Greater than 95% of both the bEnd.3 cells and ihBMECs were positive for acetylated LDL uptake (Fig 3B). Similarly, greater than 99% of both the bEnd.3 cells and ihBMECs were immuno-positive for vWF (Fig 3C). ihBMECs showed a strong and distinct junctional claudin-5 immunostaining. In the bEnd.3 cells, the claudin-5 immunostaining showed a more diffuse pattern with both junctional and cytosolic staining (Fig 3D). We saw a small but significantly higher (p<0.05) MFI of claudin-5 immunostaining in the bEnd.3 cells, attributed to both junctional and cytoplasmic claudin-5 staining, compared with the ihBMECs (Fig 3D). Human iPSC IMR90-4 cells were stained for the endothelial phenotypic markers and tight junction protein as the phenotype negative control (S1 Fig).

### Vascular endothelial phenotype, response to inflammatory stimuli, and erythrophagocytic phenotype of ihBMECs

**Fig 4:**
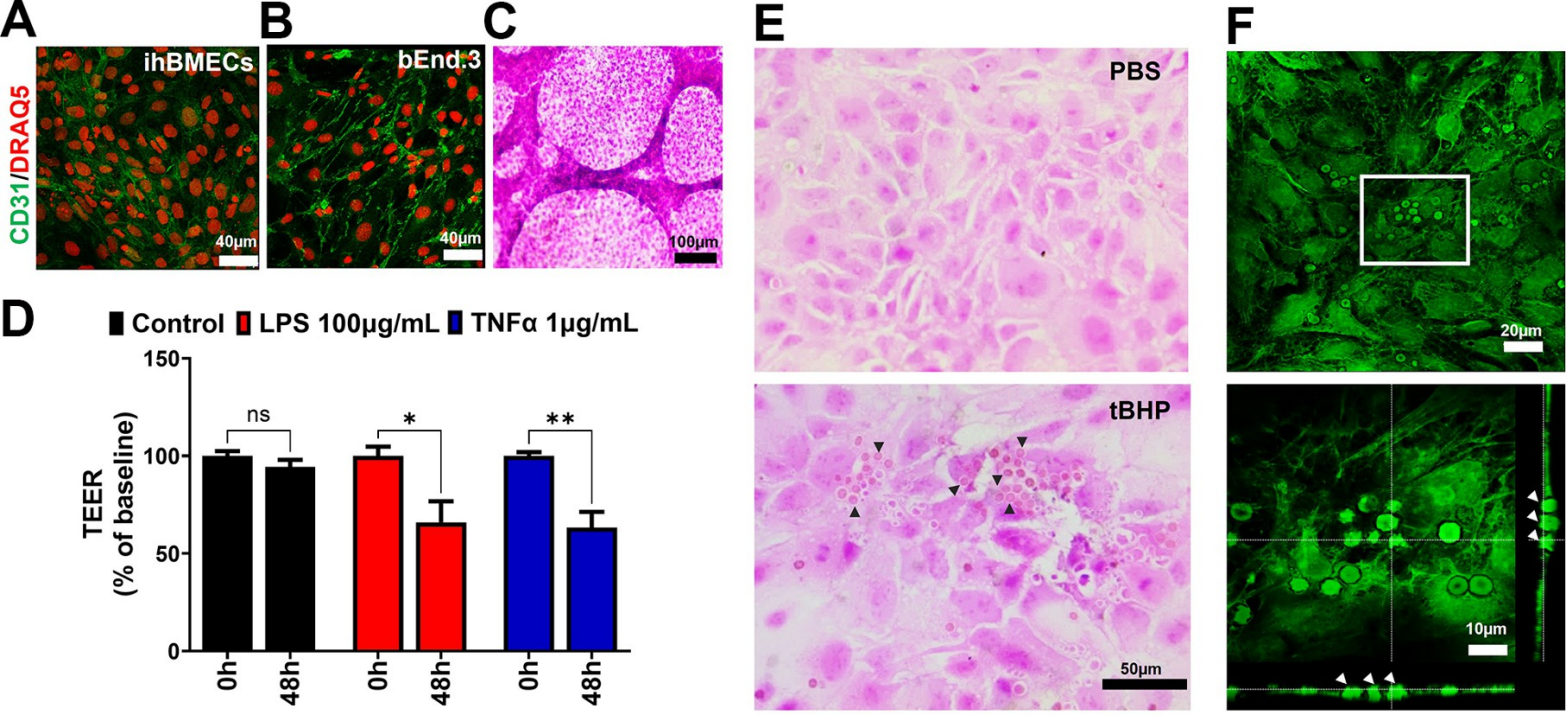
Vascular phenotype, response to inflammatory stimuli, and erythrophagocytic phenotype of ihBMECs. CD31 immunostaining in the ihBMECs (**A**) and the bEnd.3 cells (**B**). Tube-like formation by the ihBMECs grown on collagen and fibronectin coated Transwell inserts (**C**). ihBMECs respond to two inflammatory stimuli, LPS and TNFα, and show a significant reduction in TEER measurements (**D**). H&E-stained images show robust attachment and engulfment of tBHP-treated red blood cells with ihBMECs (**E**). Aged red blood cell internalization was further confirmed by maximum projection and orthogonal confocal microscopy images of H&E-stained cells showing the ihBMECs and tBHP red blood cells in autofluorescent green signal (**F**). Arrowheads show aged red blood cells internalized by the ihBMECs in E and F. Scale bar = 40 μm in A-B, 100 μm in C, 50 μm in E, and 20 μm or 10 μm in F. Data are presented as mean ± SEM of n = 3 independent repeats, and *p<0.05, **p<0.01 by two-way ANOVA and Holm-Sidak post hoc test in D.

To support the vascular endothelial phenotype of the brain endothelial monolayers, we studied the expression of the vascular endothelial marker, CD31, in the ihBMECs and bEnd.3 cells. Both brain microvascular endothelial cells expressed CD31 as shown by immunostaining (Fig 4A and 4B). The vascular endothelial phenotype of the ihBMECs was further supported by the tube-like structures spontaneously formed by the ihBMECs on collagen and fibronectin coated Transwell inserts three days after replating (Fig 4C). We further characterized the ihBMECs for their ability to respond to inflammatory stimuli, another key aspect of the brain microvascular endothelial cells [9]. Treatment of the ihBMECs with two inflammatory stimuli, LPS and TNFα, resulted in a significant reduction (~35%; p<0.05 for LPS and p<0.01 for TNFα) in the TEER (Fig 4D). To characterize the ability of ihBMECs to interact with blood cells, we studied the interactions between aged red blood cells and ihBMECs. Our results show that ihBMECs exhibit an erythrophagocytic phenotype, as seen by the H&E-stained images showing robust attachment and engulfment of aged red blood cells (tBHP-treated red blood cells) and not PBS-treated control red blood cells (Fig 4E). A maximum projection confocal image of H&E-stained slides shows autofluorescent ihBMECs and tBHP-treated red blood cells (Fig 4F top panel). An orthogonal view of the confocal image clearly shows the attachment and internalization of aged red blood cells by the ihBMECs (Fig 4F bottom panel).

## Discussion

In the current study, we compared two popular *in vitro* BBB models: the ihBMECs, which are iPSC-derived human brain microvascular endothelial cells, and the bEnd.3 cells, which are immortalized murine brain microvascular endothelial cells. The ihBMECs displayed superior barrier properties (higher TEER and significantly lower tracer permeabilities). However, both the bEnd.3 cells and the ihBMECs could discriminate between the passage of the two different molecular weight markers (sodium fluorescein and FITC-dextran), showing paracellular passage selectivity of the two monolayers. FITC-dextran permeability paralleled TEER measurements across the bEnd.3 monolayer, while sodium fluorescein permeability paralleled TEER measurements across the ihBMEC monolayer. Evaluation of key endothelial phenotypic markers confirmed the expression of vWF and uptake of acetylated LDL and the vascular endothelial marker, CD31, for both the cells. Claudin-5 expression pattern differed between these two models. We observed distinct junctional claudin-5 expression in the ihBMECs, whereas junctional claudin-5 expression was less distinct and a more diffuse cytoplasmic expression of claudin-5 was observed in the bEnd.3 cells.

A key feature of the BBB is the high resistance across the brain microvascular endothelial cells resulting in the restrictive barrier properties. As a result, ongoing efforts have been directed towards the development of *in vitro* BBB models with enhanced TEER, which is a widely used indicator of endothelial junctional tightness. Primary brain endothelial cell monocultures from different species, including mice, rats, and porcine, result in TEER values that range between 100–800 ohm·cm^2^, with the porcine-derived brain endothelial cells having higher TEER values [11, 40]. However, isolation of primary brain endothelial cells is often associated with low cell yield and loss of BBB phenotype with subsequent passaging [11]. These limitations led to the development of immortalized brain endothelial cells. In this respect, the bEnd.3 cells, which are isolated from the mouse and immortalized using the polyomavirus middle T-antigen, have been extensively used and characterized since the early 2000s for *in vitro* BBB studies [21, 22, 41–43]. However, the immortalization process of the bEnd.3 cells significantly impacts their barrier properties, which is the major limitation in using them for BBB transport studies. Accordingly, the TEER values reported with the bEnd.3 cells in our hands were low (~15 to 28 ohm·cm^2^), which is consistent with the values previously reported while using the bEnd.3 monoculture model [23, 24, 43, 44]. On the other hand, ihBMECs resulted in TEER values that were ~42- to 68-fold higher in the current study (Fig 1). Based on published reports, our ihBMEC culturing protocol uses retinoic acid to enhance the TEER [45]. Though the TEER values we achieved with the ihBMECs are lower than reported *in vivo* TEER values (~8000 ohm·cm^2^) [13], these values are higher than the threshold values of 500–900 ohm·cm^2^ above which permeability of the barrier to small and large molecular weight molecules remains unchanged [16].

Passage of different molecular weight neutral tracers across the monolayer often accompanies TEER measurements to characterize passive permeability across the BBB. In the current study, we measured the passage of two different molecular weight tracers, sodium fluorescein (MW: 376 Da) and FITC-dextran (MW: 70 kDa). Given its small size, slight changes in passive BBB permeability can be detected using sodium fluorescein, while the passage of 70kDa-FITC-dextran would require larger alterations in passive BBB permeability [12]. Consistent with the low TEER measurements across the bEnd.3 monolayer, the permeability of both the tracers was an order of magnitude higher across the bEnd.3 monolayer compared with ihBMECs (Fig 1B). The permeability of FITC-dextran was 3.4 ± 0.3 x 10^−7^ cm/s and 2.2 ± 0.4 x 10^−8^ cm/s and that of sodium fluorescein was 2.3 ± 0.06 x 10^−6^ cm/s and 3.3 ± 0.3 x 10^−7^ cm/s across the bEnd.3 cells and ihBMECs, respectively. These values are in range with previous work using the bEnd.3 cells and ihBMECs [20, 43, 46].

To look specifically at the monolayer permeability and leakiness of the brain endothelial monolayers, we compared the permeability of tracers across cell-free Transwell inserts (spontaneous migration) with the tracer permeability across the Transwell inserts in the presence of the endothelial monolayer. While Fig 1B provides information on the permeability of the tracer, Fig 1C and 1D provide information on the percentage of spontaneous (free) migration of the tracer which can be blocked by the endothelial monolayer. The spontaneous migration of FITC-dextran was significantly reduced by both the bEnd.3 and the ihBMEC monolayers by >80% and >90%, respectively (Fig 1). However, while the spontaneous migration of sodium fluorescein was efficiently blocked by the ihBMECs (> 85%), only 15% of sodium fluorescein passage was blocked by the bEnd.3 monolayer. These results show that majority of the sodium fluorescein added to the bEnd.3 monolayer migrated freely to the basolateral chamber (Fig 1). Collectively, these results suggest that while the bEnd.3 monolayer is relatively leaky to small molecular weight tracers (e.g., sodium fluorescein), permeability of large molecular weight tracers is largely restricted by the bEnd.3 monolayer.

As would be expected based on the different molecular weights, the permeability of sodium fluorescein was significantly higher than that of FITC-dextran across both the bEnd.3 and ihBMEC monolayers. This suggests that the bEnd.3 monolayer, though leakier than the ihBMECs based on low TEER, can discriminate between the passive permeability of these two different molecular weight tracers. This is consistent with previous work showing that the bEnd.3 monolayer can discriminate between the passive permeabilities of different molecular weight neutral tracers [43]. Taken together, these results suggest that despite having very low TEER compared to the ihBMECs, the bEnd.3 cells may be suitable for passive transport studies of macromolecules but not small molecules.

In the current study, we found that tracer permeability values did not always parallel TEER measurements. For example, sodium fluorescein permeability did not correlate with TEER measurements across the bEnd.3 monolayer, while FITC-dextran permeability showed a strong negative correlation with TEER measurements across the bEnd.3 monolayer (Fig 2). On the contrary, sodium fluorescein permeability was a significant inverse correlate of TEER measurements across the ihBMEC monolayer. As shown in Fig 1, the bEnd.3 monolayer allows a majority of the sodium fluorescein to pass freely and forms a leaky barrier for this small molecular weight tracer, rendering sodium fluorescein less sensitive to small TEER changes (10–30 ohm·cm^2^) across the bEnd.3 monolayer. On the other hand, the ihBMEC monolayer forms a tighter barrier, almost completely restricting the passage of FITC-dextran, which perhaps makes this tracer less sensitive to TEER changes across the ihBMEC monolayer. These results further suggest using different and appropriate molecular weight tracers to corroborate TEER measurements, which is consistent with a recent study showing that low TEER does not always correlate with high tracer permeability [40]. Notably, despite the high TEER values with the ihBMECs, tracers were still detected in the basolateral chamber. This residual leak, previously reported by others, may be attributed to the passage of free FITC (FITC detached from FITC-dextran) and some involvement of the transcellular route in sodium fluorescein passage, as suggested previously [12, 16].

High expression of tight-junction proteins such as claudin-5, zonula occludens-1, and occludin is necessary for high TEER and limited passive permeability across the BBB. Among the tight-junction proteins, claudin-5 is critical for maintaining barrier properties [47, 48], and in the current study, we compared the expression of claudin-5 in bEnd.3 cells and ihBMECs side-by-side using immunostaining. Both *in vitro* models showed strong claudin-5 expression (Fig 3). However, consistent with previous work, we found a distinct junctional rather than the cytoplasmic location of claudin-5 in the ihBMECs [14, 20], whereas both junctional and cytoplasmic claudin-5 expression was observed in the bEnd.3 cells [23]. This robust junctional localization of claudin-5 in the ihBMECs perhaps correlates with their higher TEER and reduced passive permeability, consistent with studies showing that strong junctional rather than cytoplasmic localization of claudin-5 enhances barrier properties [42]. We also studied the expression of key endothelial makers, vWF, and acetylated LDL uptake. Both models showed robust expression of vWF and uptake of acetylated LDL, confirming the endothelial phenotype of both these *in vitro* models.

This study has limitations. The data presented here largely focus on the paracellular barrier properties of the bEnd.3 and the ihBMECs. Besides limited paracellular transport, polarized expression of influx and efflux transporters regulates the transport of xenobiotics and endogenous small molecules at the BBB and is important while studying drug transport across the BBB. While we did not compare the expression of BBB transporters in the present study, the functionality of these transporters in the bEnd.3 and ihBMECs has been extensively reported [31, 37]. Parallel comparisons of transporter expression in the bEnd.3 cells and primary mouse brain endothelial cells show higher expression in the primary cells [37], and future work is needed to compare the transporter expression between the bEnd.3 cells and ihBMECs. Additionally, since brain microvascular endothelial cells are vascular cells, characterizing the vascular phenotype of these cells is crucial while differentiating them from similar cells (epithelial cells) that also exhibit limited paracellular transport properties and tight-junction protein expression. In our hands, both the ihBMECs and bEnd.3 cells express CD31, a vascular endothelial marker. Interestingly, the ihBMECs also showed spontaneous tube-like formation, suggesting a vascular endothelial phenotype (Fig 4). However, we did not study the epithelial signature of the ihBMECs. Recent studies show a reduction or complete absence of vascular endothelial markers in ihBMECs and show the expression of epithelial junctional proteins [9, 49]. This has led to the suggestion that ihBMECs exhibit a mixed endothelial-epithelial gene expression profile [9, 19]. Besides use for transport studies, *in vitro* BBB models are extensively used to study the interactions of blood cells with the brain microvasculature. For example, immune cell interactions with and trafficking across the brain microvascular endothelial cells are restricted at the BBB and the brain microvascular endothelial cells respond to inflammatory stimuli through upregulation of adhesion molecules and increased barrier permeability [50, 51]. While we have not studied the expression of adhesion molecules, we examined the response of the ihBMECs to two different inflammatory stimuli known to increase paracellular permeability across the brain endothelial monolayer [50]. Both LPS and TNFα significantly reduced TEER measurements, suggestive of a leakier ihBMEC barrier in response to inflammatory stimuli (Fig 4). To date, only one study reports the use of ihBMECs to study immune cell trafficking [52], and no study has reported the use of ihBMECs to study brain microvascular endothelial-red blood cell interactions. The latter is relevant given the increasing body of literature showing that vascular endothelial cells can act as unprofessional phagocytes and internalize a variety of cargo, including blood clots [30], pathogenic bacteria [25], and red blood cells [26–29]. All the studies demonstrating the phagocytic phenotype of the vascular endothelium were done using immortalized cells lines or *in vivo* rodent models. Our previous work, along with other studies, shows that the bEnd.3 cells [27, 32] and HCMEC/D3 human brain microvascular endothelial cells [28] exhibit an erythrophagocytic phenotype for aged/damaged red blood cells. Since the red blood cell adhesion and phagocytic repertoire of the ihBMECs had not been reported, we determined the erythrophagocytic phenotype of the ihBMECs. In accordance with the reported phagocytic phenotype of vascular endothelial cells, including the brain microvascular endothelial cells, our results show that ihBMECs also demonstrate cell adhesion and phagocytic characteristics (Fig 4). This is the first study to show that the ihBMECs recapitulate the erythrophagocytic phenotype shown by the bEnd.3 cells [27, 32] and human brain microvascular cells [28], making them a suitable *in vitro* model to study brain microvascular endothelial cell-red blood cell interactions.

## Conclusions

In conclusion, both the murine immortalized cell line, bEnd.3 cells, and the iPSC-derived human brain endothelial cells, ihBMECs, express key endothelial phenotypic markers, show red blood cell adhesion and phagocytic features, and express vascular endothelial markers, making them useful models for BBB mechanistic studies. The major difference between these two *in vitro* BBB models was in their paracellular barrier characteristics, with the ihBMECs having significantly higher TEER and restrictive permeability to the small molecular weight tracer (sodium fluorescein). However, despite the many-fold higher TEER measurements across the ihBMECs, the bEnd.3 monolayer restricted > 80% passage of free FITC-dextran. Our results, therefore, suggest that the bEnd.3 cells may be a suitable model for transport studies of large molecules and for cell interaction and migration studies despite their low TEER [27]. Modification of the culture conditions, e.g., serum-free conditions and co-cultures, can be adapted to further enhance bEnd.3 barrier properties [42]. Our results also suggest the need to corroborate TEER measurements with different molecular weight tracers as a more reliable measure of barrier properties for an *in vitro* BBB model designed for paracellular transport studies. Although the choice of the *in vitro* BBB model selected will ultimately be based on the nature of the investigation, the choice will also be driven by other factors such as batch-to-batch variability, time and cost of culturing, and questions about the endothelial lineage with the ihBMECs [18, 20], versus the ease of culturing, consistency between passages and commercial availability of the bEnd.3 cells.

## Supporting information

S1 Fig Negative control (iPSC) for endothelial phenotype confirmation. Phase-contrast images showing the typical colony morphology of iPSCs (A, scale bar = 250 μm). Uptake of AlexaFluor-488 labeled acetylated low-density lipoprotein (acLDL) was not detected in iPSC (B). No immunofluorescent detection of Von Willebrand factor (vWF; C) and claudin-5 (D) in iPSC. The nucleus is stained with DRAQ5 (shown as red). Scale bar = 40 μm.
(TIF)
