# Supplementary figures and images for: Comparative studies between the murine immortalized brain endothelial cell line (bEnd.3) and induced pluripotent stem cell-derived human brain endothelial cells for paracellular transport
Source: PLoS One. 2022 May 25;17(5):e0268860. doi: 10.1371/journal.pone.0268860 (PMC9132315; doi:10.1371/journal.pone.0268860)

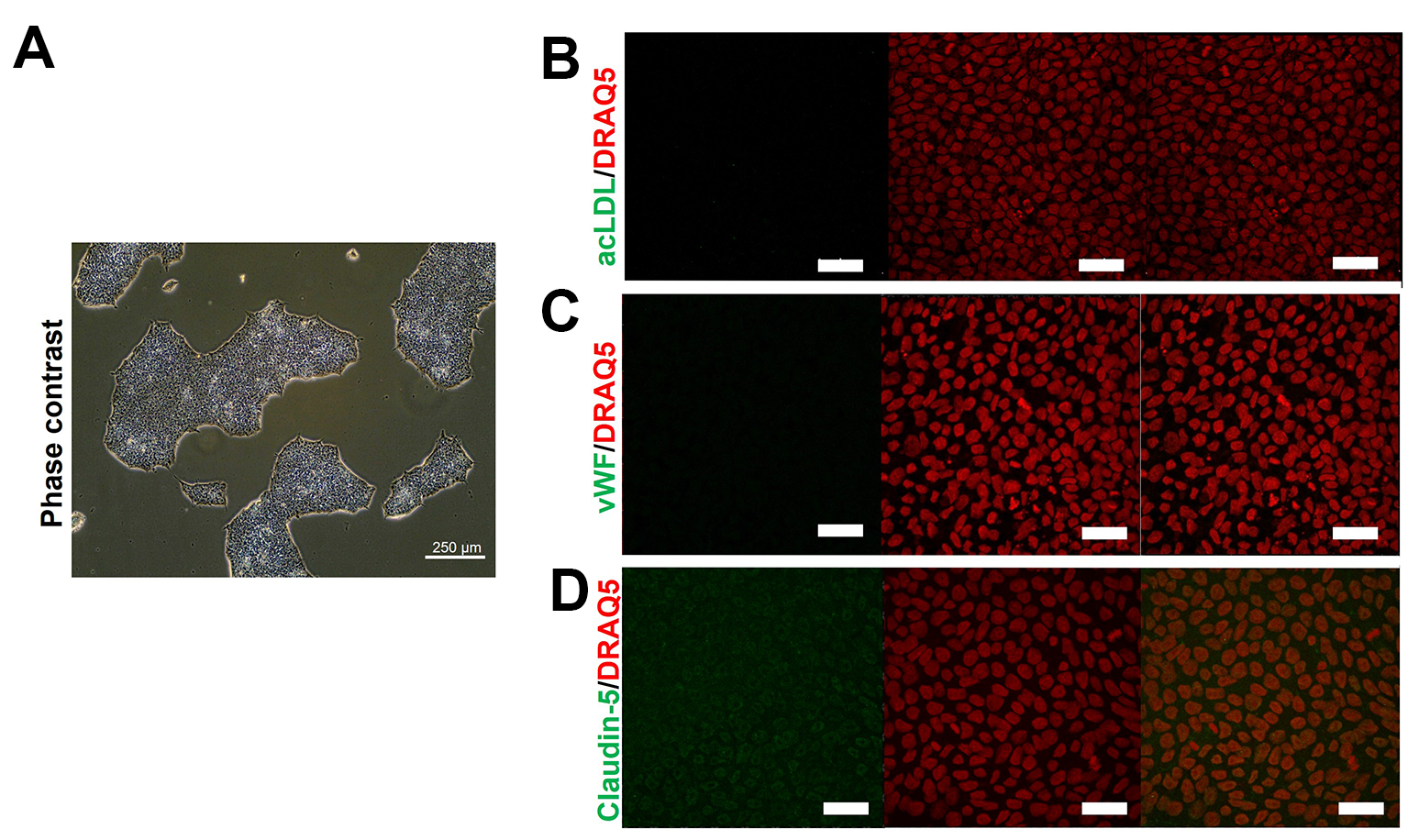

Supplement: S1 Fig — Phase-contrast images showing the typical colony morphology of iPSCs (A, scale bar = 250 μm). Uptake of AlexaFluor-488 labeled acetylated low-density lipoprotein (acLDL) was not detected in iPSC (B). No immunofluorescent detection of Von Willebrand factor (vWF; C) and claudin-5 (D) in iPSC. The nucleus is stained with DRAQ5 (shown as red). Scale bar = 40 μm. (TIF) [file pone.0268860.s001.tif]
